# Protocol to quantify LRRK2-mediated Rab10 phosphorylation using high-content imaging

**DOI:** 10.1016/j.xpro.2026.104819

**Published:** 2026-09-04

**Authors:** Beatrice Masotti, Paolo Lorenzon, Giuseppina Covello, Elisa Greggio

**Affiliations:** 1 Department of Biology, University of Padova, Padova, Italy; 2 San Camillo Hospital, IRCCS, 30126 Venice, Italy

**Keywords:** Cell Biology, Molecular Biology, Neuroscience

## Abstract

Rab10 phosphorylation at Thr73 (pRab10) is a well-established readout of the kinase LRRK2, a protein whose mutations are associated with Parkinson’s disease. Here, we present a protocol for high-content quantification of endogenous pRab10-positive vesicles in primary astrocyte-enriched cultures using the Operetta CLS system. We describe steps for cell culture, immunofluorescence, image acquisition, and quantitative analysis using Harmony software. This protocol is applicable to other LRRK2-expressing cells and offers a scalable platform for quantitative studies of LRRK2 physiological and pathological activity.

## Highlights

• Guidance for detection of LRRK2 activity in LRKK2-expressing cells
• Instructions for pRab10 immunostaining in primary astrocyte-enriched cultures
• Steps for automated quantification of pRab10-positive vesicles using Harmony software

## Abstract (editor highlights)

Publisher’s note: Undertaking any experimental protocol requires adherence to local institutional guidelines for laboratory safety and ethics.

## Graphical abstract

**Figure:**
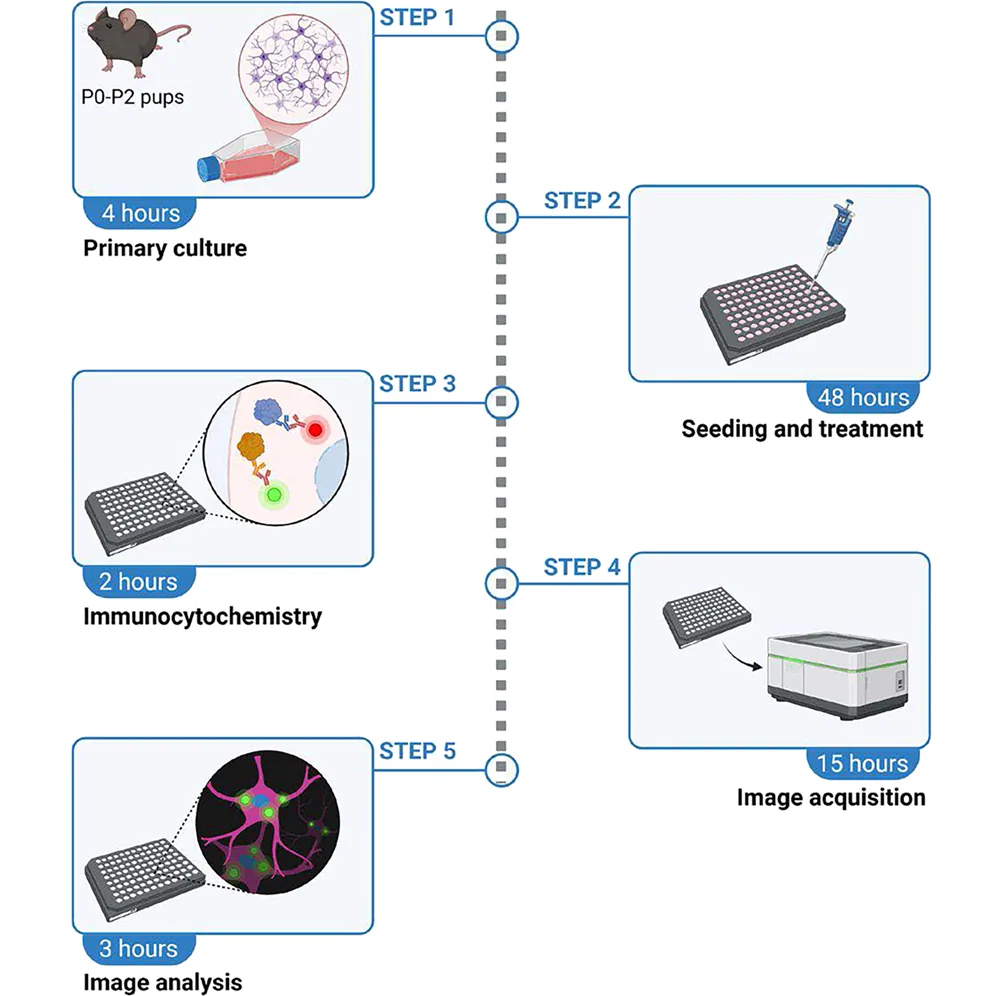

## Before you begin

The kinase Leucine-rich repeat kinase 2 (LRRK2) is an attractive target in Parkinson’s disease (PD) research, as its disease-associated hyperactivity represents a compelling therapeutic avenue.^1^^,^^2^ LRRK2 phosphorylates a subset of Rab GTPases within a highly conserved region of the Switch II domain.^3^ Rab10 is one of the best-validated LRRK2 substrates, and phospho-specific antibodies recognizing the LRRK2 phosphorylation site at Thr73 (e.g., MJF-R21 clone) are highly specific and are widely used as a readout of LRRK2 kinase activity in cell-based systems, animal models, and human samples.^4^^,^^5^ LRRK2-mediated phosphorylation of Rab10 occurs at lysosomes and other endocytic vesicles,^6^^,^^7^ generating well-defined punctate structures amenable for immunocytochemistry analysis.^8^ Here, we describe the steps for imaging and quantification of pRab10-positive vesicles in primary cortical astrocyte-enriched glial cells. Cells are isolated from mice carrying the *Lrrk2*^G2019S/G2019S^ mutation,^9^ the most common LRRK2 variant associated with both familial and sporadic PD,^10^ although the protocol can also be applied to wild-type (WT) cultures or adapted to cells carrying other LRRK2 mutations. In this protocol, we provide a step-by-step guide to preparing primary astrocyte-enriched glial cultures and performing subsequent immunostaining for pRab10-positive vesicles, nuclei, and F-actin using phalloidin as a cytoskeletal marker. Image acquisition is performed using the Operetta CLS high-content imaging system, followed by automated image analysis using Harmony software to obtain a precise quantification of the number of vesicles per field. This protocol exploits the acquisition of multiple fields per well, enabling statistically robust detection and quantification of pRab10 levels. The assay supports high-throughput testing of compounds that modulate LRRK2 kinase activity, including estimation of cellular IC_50_ values, as demonstrated using the highly selective LRRK2 inhibitor MLi-2.^11^ Moreover, the protocol enables detection of changes in pRab10 levels induced by modulators of LRRK2 signaling pathways, including lysosomal stress. This is illustrated using the lysosomal stressor L-Leucyl-L-Leucine-O-methyl ester (LLOME), which has been shown to increase LRRK2-mediated Rab10 phosphorylation.^12^^,^^13^ Automated nuclear counting provides an internal measure of cell viability, facilitating evaluation of tool compounds or pathway modulators without confounding toxicity effects.

### Preparation of primary cortical astrocyte-enriched glial cultures from murine pups

**Figure 1:**
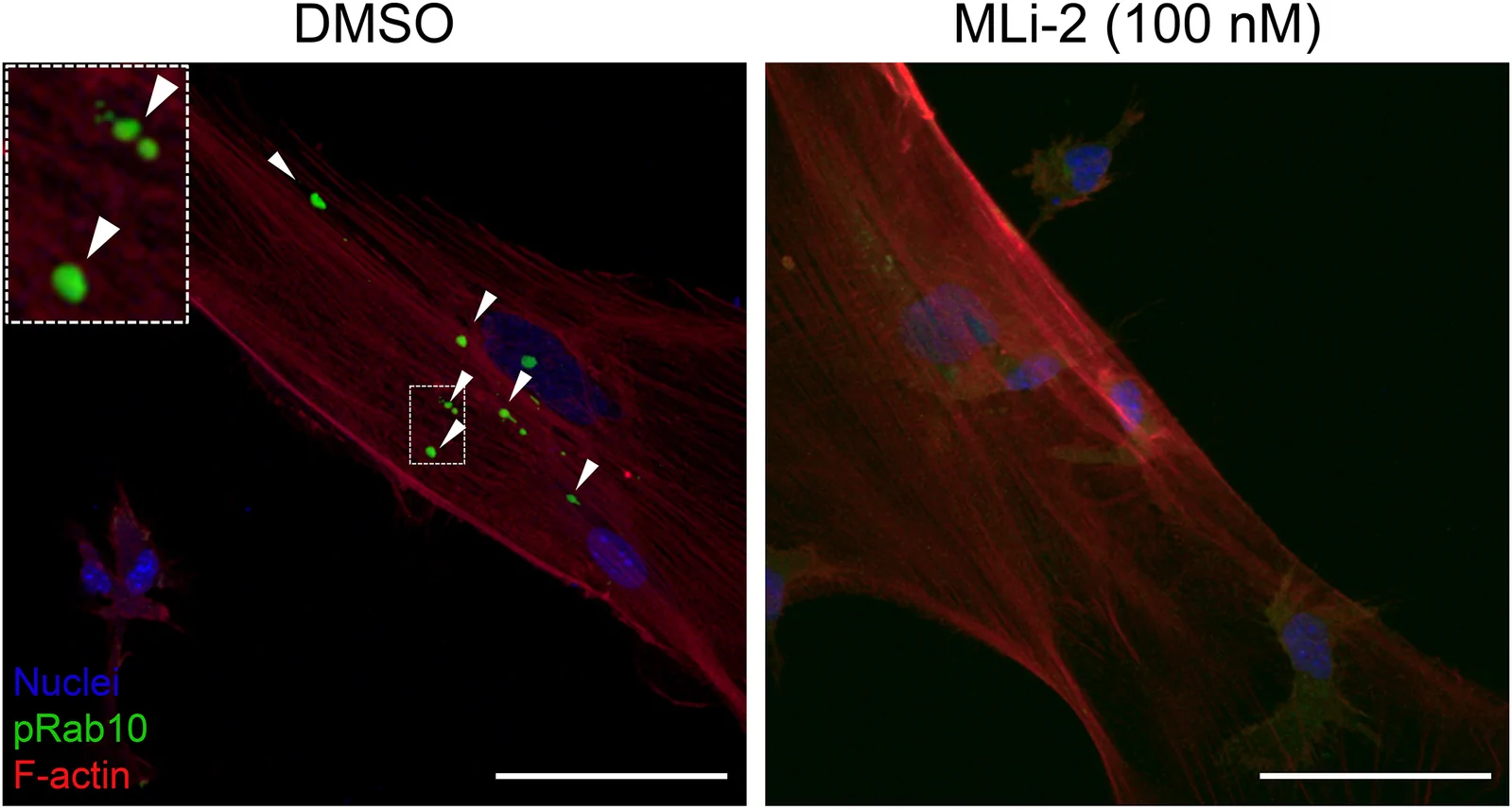
Representative images of pRab10-positive vesicles in primary astrocyte-enriched cultures Cells carrying the endogenous *Lrrk2* G2019S mutation were treated with vehicle control (DMSO) or the LRRK2 kinase inhibitor MLi-2 (100 nM, 24 h) and imaged using a Zeiss LSM 900 Confocal Microscope with a 40× objective. pRab10-positive vesicles are visualized by immunofluorescence staining. Scale bar: 50 μm.

***Note:*** We prepare primary cortical astrocyte-enriched glial cultures from homozygous *Lrrk2*^G2019S/G2019S^ knock-in mice, in which the endogenous murine *Lrrk2* locus carries the G2019S substitution at the conserved residue corresponding to the human pathogenic *LRRK2* G2019S mutation.^14^ These cultures yield a well-characterized and relevant model with clear and specific signal of pRab10 clusters (Figure 1), enabling medium- to high-throughput screening applications. In addition, pRab10-positive structures detected in these cultures show association with lysosomal compartments (Figure S1), consistent with previous reports linking LRRK2-dependent Rab10 phosphorylation to lysosomal pathways.^7^^,^^15^

Although this preparation does not yield purified astrocytes, the protocol is based on standard neonatal cortical glial culture conditions that favor the expansion of adherent astrocytic cells.^16^^,^^17^ Accordingly, the resulting cultures should be considered primary cortical astrocyte-enriched glial cultures. Because the protocol does not include a dedicated astrocyte-purification step or specific depletion of microglia and oligodendrocyte-lineage cells, other glial populations may be present to variable extents across preparations. We therefore recommend assessing culture composition using cell-type-specific markers when cell-type purity is critical for downstream applications. Since LRRK2 activity is functionally characterized and pathologically relevant in glial cells, including astrocytes and microglia, these cultures represent a useful primary brain-cell model for studying LRRK2-dependent Rab10 phosphorylation.^17^^,^^18^^,^^19^ In addition, this protocol can be readily applied to astrocytes or other glial cells derived from different brain regions (e.g., striatum) or other LRRK2-expressing cells.

1. Cortices dissection:
  a. Behead postnatal pups (P0-P2) and excise the brain, lifting from the cerebellum to the olfactory bulbs. Place the brain in a cell culture dish containing cold Dulbecco’s Phosphate-Buffered Saline (DPBS) supplemented with 1% Penicillin–Streptomycin (P/S).
  b. Under a dissecting microscope, carefully separate the cortices and move them to a new dish containing cold DPBS supplemented with 1% P/S.
2. Tissue dissociation and primary glia isolation:
  a. Work inside a biological hood. Using a pipette, remove DPBS from the cell culture dish without aspirating the tissue.
  b. Add 1 ml of room temperature (RT) Dulbecco’s Modified Eagle Medium (DMEM) supplemented with 1% P/S and mechanically homogenize tissue by gently pipetting up and down 10–15 times with a P1000 pipette.
  c. Place a 70 μm cell strainer on top of a 50 mL tube, move the homogenized tissue onto the strainer. Rinse the cell culture dish with 1 ml of DMEM and transfer the remaining tissue to the strainer.
  d. Sift the tissue using a syringe plunger to facilitate the passage of cells through the strainer.
  e. Add 1 ml of DMEM to wash the strainer thoroughly, then remove the strainer and add 22 ml of DMEM to reach a final volume of 25 ml in the falcon tube.
  f. Centrifuge 15 min at 300 × *g* at RT and discard the supernatant.
  g. Wash the pellet twice: discard the supernatant, resuspend the pellet in 25 ml of DMEM, then centrifuge 15 min at 300 × *g* at RT.
  h. Resuspend the pellet in 1 ml of DMEM, supplemented with 1% P/S and 10% Fetal Bovine Serum (FBS).
  i. Seed 5∗10^6^ cells/10 ml medium in the appropriate flask (3 pups correspond to a T75 flask) and incubate at 37°C with 5% CO_2_.
  j. After 7 days, change the medium completely and replace it with fresh DMEM supplemented with 1% P/S and 10% FBS.
  k. After 12–15 days in vitro (DIV), astrocyte-enriched glial cultures reach 80% confluency and are ready for experiments.

### Innovation

This protocol provides a complete workflow for detecting and quantifying Rab10 phosphorylation, a well-established readout for LRRK2 kinase activity, in primary cortical cultures. Rather than introducing a new imaging platform, the value of this protocol lies in the standardization and validation of a scalable high-content imaging pipeline that enables reproducible detection, segmentation and quantification of endogenous pRab10-positive vesicular structures in a disease-relevant primary cellular model carrying the *Lrrk2*^G2019S/G2019S^ mutation.

A key strength of the protocol is that it translates a biologically relevant LRRK2 activity readout into a medium-to high-throughput format suitable for systematic pharmacological or pathway-modulation studies. The workflow combines optimized culture preparation, immunostaining, automated multi-field image acquisition and a detailed Harmony-based image-analysis pipeline for vesicle detection and classification. This reduces operator-dependent bias and enables quantitative comparison across treatment conditions, including LRRK2 kinase inhibition and lysosomal stress paradigms.

Because the assay detects endogenous pRab10 signal without requiring LRRK2 or Rab10 overexpression, it provides a physiologically relevant platform for studying LRRK2 signaling in primary glial cultures. In addition, the modular analysis strategy may be adapted to other LRRK2- and Rab10-expressing cellular models, including immortalized cell lines (e.g., RAW 264.7 macrophages or A549 cells),^8^^,^^15^ induced pluripotent stem cell-derived macrophages and microglia,^20^ or other primary cell types, provided that staining, acquisition, and segmentation parameters are appropriately optimized.

### Institutional permissions

Housing and handling of mice were done in compliance with national guidelines. All procedures performed with mice were approved by the Ethical Committee of the University of Padova and the Italian Ministry of Health (license 1005/2024-PR and D2784.N·KOC).

## Key resources table

**Table:** 

REAGENT or RESOURCE	SOURCE	IDENTIFIER
**Antibodies**		

Anti-RAB10 (phospho T73) MJF-R21 (dilution 1/500)	Abcam	Cat# ab241060
Alexa 488 rabbit (dilution 1/500)	Invitrogen	Cat# A11034
Phalloidin-iFluor 647 reagent (dilution 1/1,000)	Abcam	Cat# ab176759
Hoechst (1:10,000)	Invitrogen	Cat# H3569

**Chemicals, peptides, and recombinant proteins**		

DPBS	Biowest	Cat# L0615
DMEM	Biowest	Cat# L0103
FBS	S. I. A. L. Group	Cat# yourSIAL-FBS-SA
Penicillin – Streptomycin	Biowest	Cat# L0022
Trypsin	Gibco	Cat# 15090046
Sodium Azide (NaN_3_)	Sigma-Aldrich	Cat# S2002
Paraformaldehyde 16% (PFA)	TermoFisher	Cat# 043368-9M
MLi-2	Tocris (Biotechne)	Cat# 5756
LLOME	Sigma-Aldrich	Cat# L7393
Dimethyl sulphoxide (DMSO)	Carl ROTH	Cat# RTA994.2
Triton X-100	Sigma-Aldrich	Cat# 9002931

**Experimental models: Organisms/strains**		

B6.Cg-*Lrrk2*^*tm1.1Hlme*^/J mice, 3–6 months old, male	The Jackson Laboratory	Strain# 030961
C57BL/6J mice, 3–6 months old, male	The Jackson Laboratory	Strain# 000664

**Software and algorithms**		

Harmony™	Revvity	N/A
GraphPad Prism (v. 10.4.2)	GraphPad Software	RRID:SCR_002798

**Other**		

μClear® 96-well (CELLSTAR®)	Greiner Bio-One	Cat# 655090
Operetta CLS™	Revvity	N/A

## Materials and equipment

**Table:** PFA 4%

Reagent	Final concentration	Amount
PFA 16%	4%	10 mL
DPBS	N/A	30 mL
**Total**	**N/A**	**40 mL**

Store aliquots at −20°C for up to 6 months. Thaw immediately before use. Do not refreeze after thawing.

**Table:** Permeabilization solution

Reagent	Final concentration	Amount
Triton X-100	0.1%	50 μL
DPBS	N/A	49.95 mL
**Total**	**N/A**	**50 mL**

Prepare the solution just before use.

**Table:** Blocking solution

Reagent	Final concentration	Amount
FBS	5%	2.5 mL
DPBS	N/A	47.5 mL
**Total**	**N/A**	**50 mL**

Prepare the solution just before use.

## Step-by-step method details

### Cell seeding and treatment

**Timing: 2 days**

This section describes the steps for seeding and treatment of astrocyte-enriched primary cultures prior to staining and automated imaging and quantification of pRab10-positive vesicles.

1. When astrocyte-enriched cells in the flask reach 80% confluency, seed 50,000 cells per well in a black-walled, clear-bottom, 96-well plate with lid.
  **CRITICAL:** Avoid seeding cells in outer wells (recommended layout shown in Figure 2).
  a. Technical limitation: The 40× objective lens cannot reliably acquire external wells images due to steric hindrance;
  b. Edge effects: Evaporation, temperature fluctuations, and uneven humidity can introduce variability in cell behavior or staining intensity, leading to unreliable or inconsistent data.
    Fill outer wells with DPBS.
    ***Note:*** After 48 h, cells reach confluency and can be treated with the appropriate compound.

**Figure 2:**
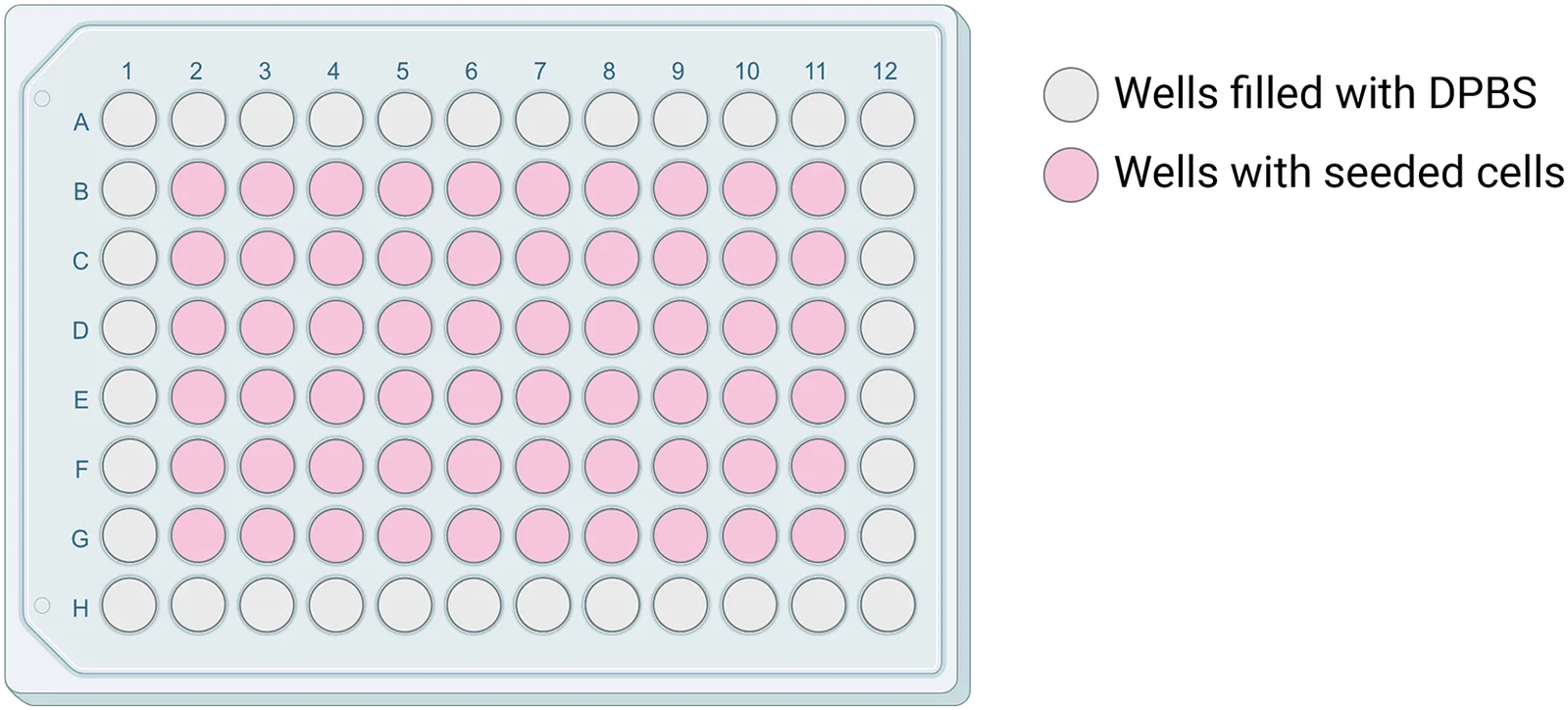
Recommended layout of a 96-well plate for Operetta CLS image acquisition
2. Treat cells with the appropriate compound.
  a. For MLi-2 treatment: remove culture medium and treat cells with fresh DMEM containing MLi-2 at the desired concentration for 90 min.
    To obtain a dose-response curve, prepare serial dilutions starting at 1 μM and dilute 1:5 across wells.
  b. For LLOME treatment: remove culture medium and treat cells with fresh DMEM containing either MLi-2 (100 nM) or LLOME (1 mM) for 2 h. To assess LRRK2-dependence of LLOME-induced Rab10 phosphorylation, co-treat cells with LLOME and MLi-2 (1 mM and 100 nM respectively) for 2 h.
    The LLOME concentration was selected as described by Bonet-Ponce et al.^12^
    ***Note:*** A schematic of the treatment workflow is shown in Figure 3.
    ***Note:*** MLi-2 is dissolved in DMSO. As a control, include a vehicle-only condition containing the same amount of DMSO as used for the highest MLi-2 concentration.

**Figure 3:**
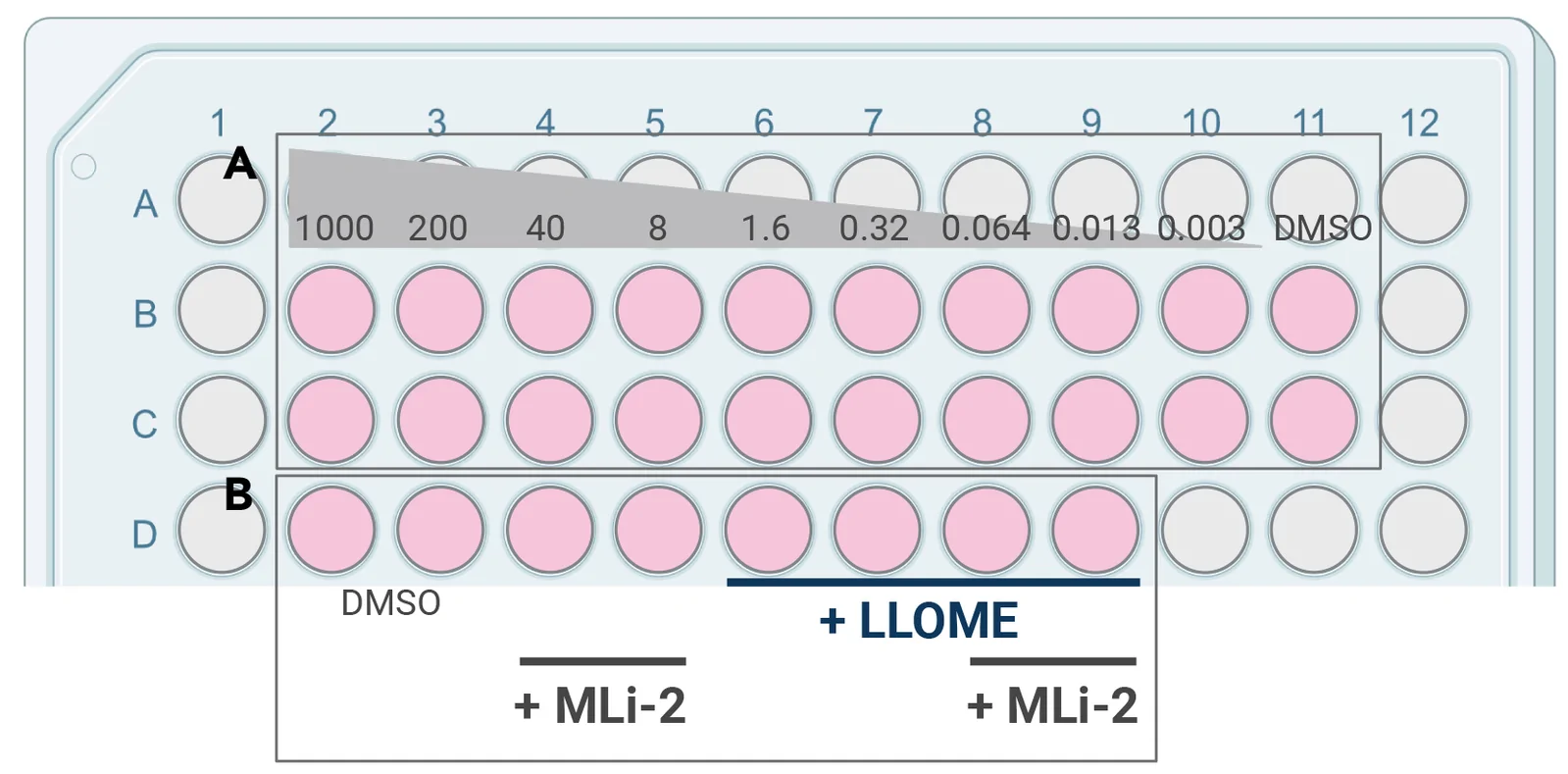
Experimental layout for MLi-2 dose-response curve and LLOME treatment (A) MLi-2 serial dilutions (1:5; concentrations expressed in nM). (B) Treatment conditions in row D: DMSO (wells D2–D3), MLi-2 (100 nM, wells D4–D5), LLOME (1 mM, wells D6–D7), or LLOME co-treated with MLi-2 (wells D8–D9).

### Cell fixation and staining with primary antibody

**Timing: 16–18 h (2 h steps 3–8, plus overnight incubation)**

This section describes the fixation, permeabilization, blocking, and primary antibody staining of astrocyte-enriched primary cultures.

3. Remove medium and fix cells with 4% paraformaldehyde (PFA) in DPBS (100 μL per well) for 20 min at RT.

**CRITICAL:** To avoid disrupting cell integrity, dispense the PFA gently along the edge of each well. From this step onward, use a multichannel pipette and a reagent reservoir.

4. Carefully remove PFA using a multichannel pipette, aspirating from the edge of each well to avoid detaching cells.
5. Wash with DPBS 5 times. Use a multichannel pipette to remove DPBS during the first wash, then add fresh DPBS and discard by inverting the plate.

***Note:*** Work inside a chemical fume hood when performing PFA fixation and washes.

**CRITICAL:** Thoroughly remove residual PFA to minimize background fluorescence.

**Pause point:** After washes, fixed cells can be stored in DPBS containing 0.05% sodium azide (NaN_3_) tightly sealed at 4°C, for up to 2 weeks. Avoid prolonged storage (>2 weeks).

6. Permeabilize cells with Permeabilization solution (100 μL per well) for 20 min at RT.
7. Block with Blocking solution (100 μL per well) for 1 h at RT.
8. Incubate cells with primary antibody diluted 1:500 (v/v) in Blocking solution (40 μL per well) at 4°C overnight (12–16 h).

**CRITICAL:** During steps 7–8, place plates on several layers of moistened absorbent paper inside a lidded polystyrene box to maintain a humid environment and prevent sample drying.

### Cell staining with secondary antibody

**Timing: 2 h**

This section describes the final staining steps, including secondary antibody, phalloidin, and nuclear staining.

9. Wash cells 5 times with DPBS.
10. Incubate cells with fluorescently conjugated secondary antibody and phalloidin diluted in DPBS (40 μL per well) for 1 h at RT (goat anti-rabbit Alexa Fluor 488, 1:500; phalloidin Alexa Fluor 647, 1:1,000).

***Note:*** From this step onward, protect samples from light.

11. Wash cells 5 times with DPBS.
12. For nuclear staining, incubate cells with Hoechst diluted 1:10,000 in DPBS (100 μL per well) for 5 min at RT.
13. Wash cells 3 times with DPBS.
  a. Leave plates in DPBS supplemented with 0.05% NaN_3_.

**Pause point:** Once stained, cells can be stored in DPBS containing 0.05% NaN_3_, tightly sealed and protected from light at 4°C for up to 3 weeks. However, imaging immediately after staining is recommended for optimal results.

### Operetta CLS image acquisition

**Timing: ∼15 h**

This section describes image acquisition using the Operetta CLS high-content imaging system, including channel selection and acquisition parameters.

14. Analyze plates using Operetta CLS high-content imaging system equipped with a 40× water-immersion objective.
  a. For detection of Hoechst, use excitation at 355–385 nm and emission at 430–500 nm.
  b. For detection of Alexa Fluor 488, use EGFP channel with excitation at 460–490 nm and emission at 500–550 nm.
  c. For detection of Alexa Fluor 647, use excitation at 615–645 nm and emission at 655–760 nm.

**Figure 4:**
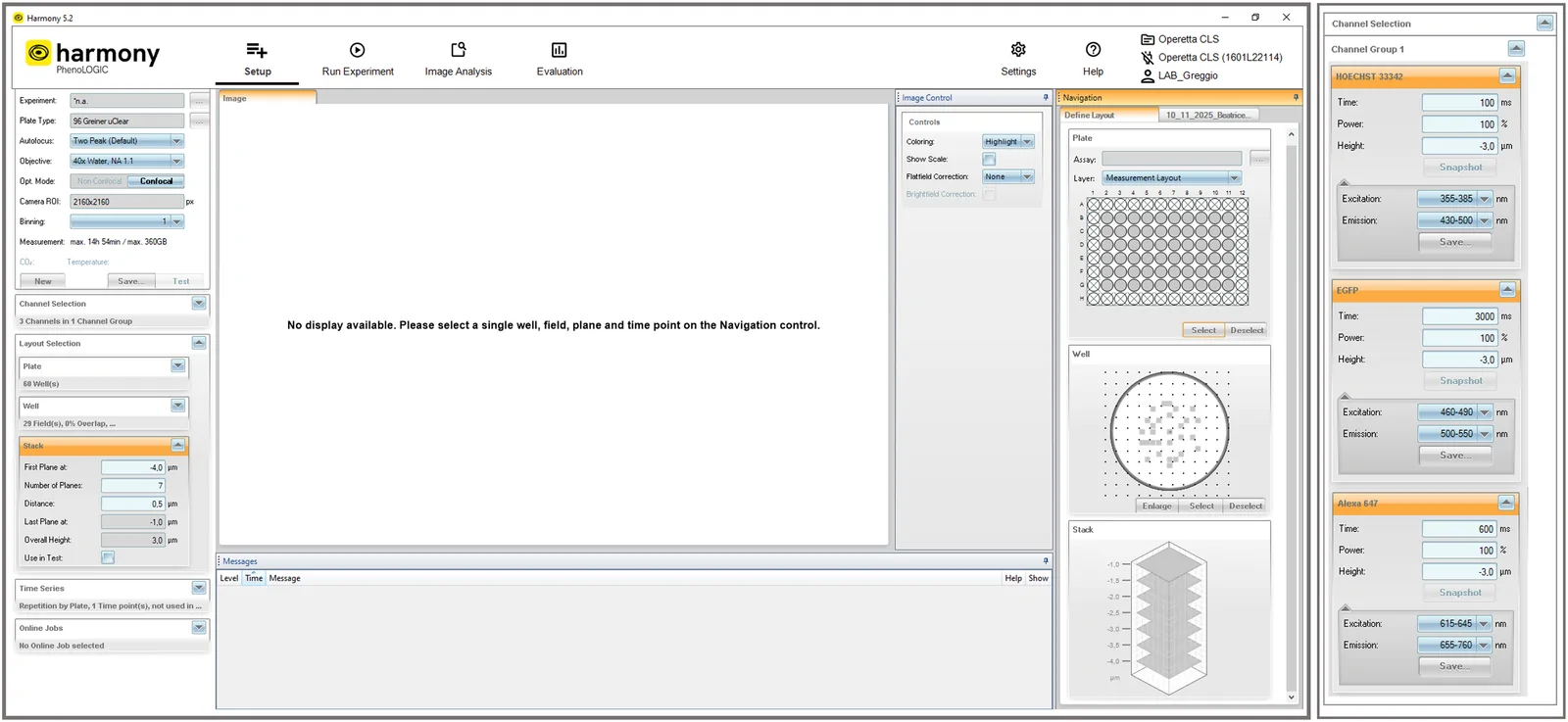
Illustration of the image acquisition interface The number and depth of Z-stack planes are shown on the left, and all channel parameters are shown on the right.

***Note:*** Detailed acquisition parameters are reported in Table 1 and Figure 4.

***Note:*** Water immersion is maintained using the integrated automated water collar system. Distilled water is automatically dispensed to the objective, and excess liquid is removed throughout image acquisition, allowing consistent imaging conditions without manual intervention.

15. Start image acquisition.

***Alternatives:*** Other high-content imaging platforms with comparable capabilities (e.g., automated fluorescence imaging and analysis using appropriate objectives) may also be used; however, these were not evaluated in this study.

**CRITICAL:** Perform a test acquisition before starting the experiment to verify that imaging parameters (e.g., number of Z-stack planes) are appropriate for the cell density and cell type. Do not vary exposure time or signal intensity settings between experiments.

***Note:*** Images are acquired from 29 fields per well. Field locations are randomly selected and distributed across the well area. When necessary, field distribution may be adjusted for individual experiments (e.g., when cells are preferentially localized toward the well borders).

**Table 1:** Summary of image acquisition parameters

Object type	Experiment
Plate Type	96 Greiner μClear
Plate Format	96
Channel	Alexa 647
Channel	EGFP
Channel	HOECHST 33342
Excitation	355–385
Excitation	460–490
Excitation	615–645
Emission	430–500
Emission	500–550
Emission	655–760
Experiment	Measurement of pRab10-positive vesicles in primary astrocyte-enriched cells
Optical Mode	Confocal
Objective	40× Water, NA 1.1
Magnification	40
Number of Fields	29
Number of Planes	7
Number of Wells	60
Number of Timepoints	1
Break in Time Series	No
Signature	-
Channel Type	Fluorescence
Instrument Type	Operetta CLS
Binning	1
Repetition Mode	Plate
Number of Sequences	1
Camera ROI	2160×2160

Parameters correspond to the experiment summary table generated by the Operetta CLS software.

### Harmony software image analysis

**Timing: 3 h per plate (step 17)**

This section describes the image analysis workflow using Harmony software, including identification and quantification of pRab10-positive vesicles.

**Figure 5:**
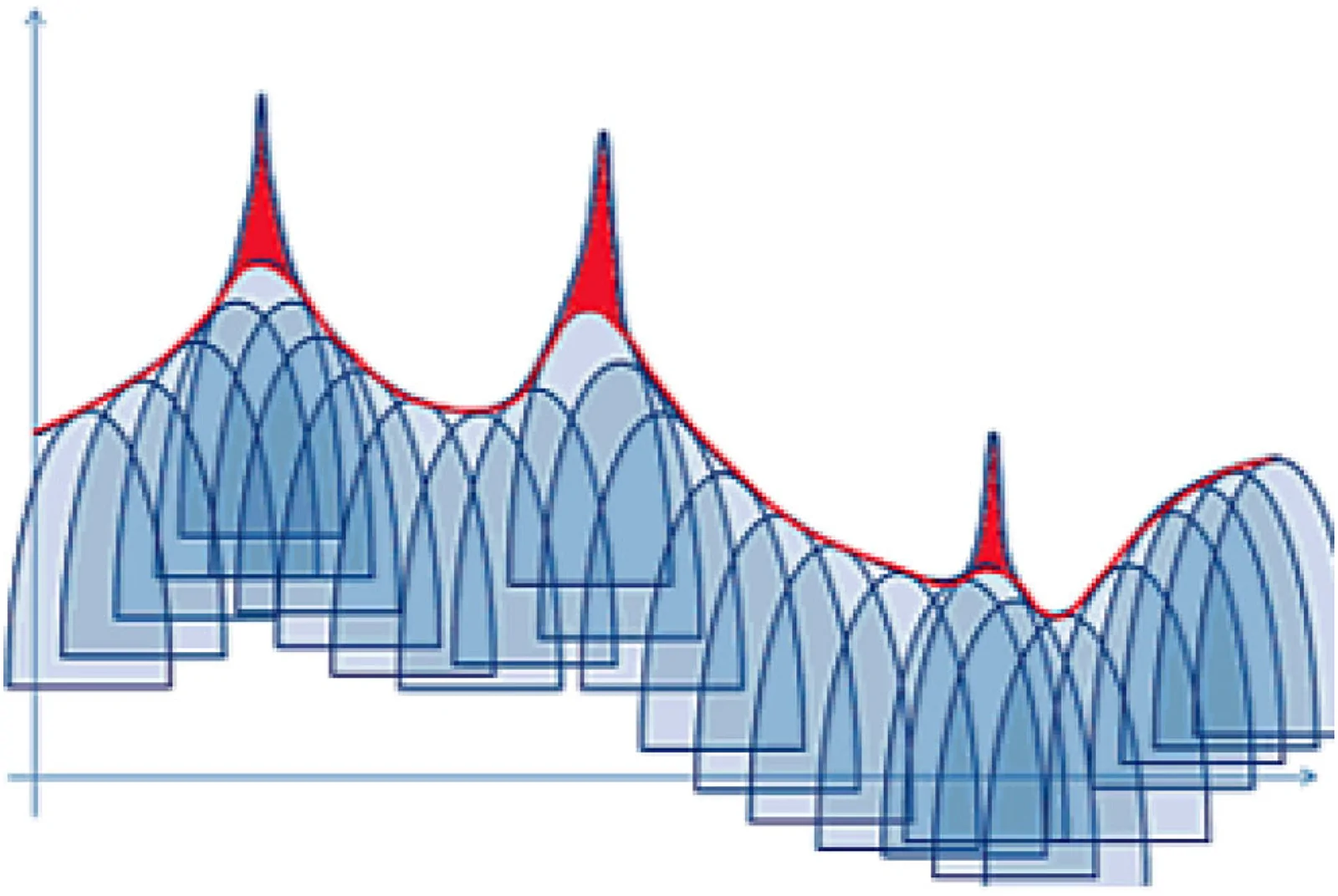
Principle of the Sliding Parabola method Image intensity is shown on the y-axis. High-intensity spots (red) are separated from smooth background (blue parabola). The filtered output is the intensity of the spot (red) on a flat background.
    ***Note:*** Optimal curvature depends on object intensity and size. In 2D analysis, the “Tune Parameter” function can help determine the most suitable curvature.
  f. Select *Find Image Region* block to identify EGFP-positive spots. Set a common intensity threshold to exclude spots near background intensity. Use *Find spots* block (Method B) to define individual spots for subsequent property calculations.
    ***Note:*** Method B parameters (detection and splitting sensitivity) depend on cell type and spot characteristics.
  g. Use *Calculate properties* block to define area, roundness, perimeter, width, length and ratio width/length (W/L). Select spots with W/L ratio >0.2 as the output population.
  h. Use *Calculate properties* block to calculate intensity of the selected population. Define a background region around the spot using Method: Resize Region (outer border −95%). The background intensity is automatically calculated, and a “Outer Broder Region” is generated for background subtraction.
  i. Use *Calculate Properties* with Method: Filter by Property to classify the population of vesicles.
    ***Note:*** Vesicles are selected according to their roundness and mean signal intensity. Vesicles meeting both criteria are retained (shown in green), while those that do not meet both criteria are discarded (shown in red).
  j. Classify vesicles by area using *Select Population* into 4 categories:
    i. Spot area < 0.3 μm^2^
    ii. 0.3 μm^2^ < Spot area < 0.8 μm^2^
    iii. 0.8 μm^2^ < Spot area < 3.5 μm^2^
    iv. Spot area > 3.5 μm^2^
      ***Note:*** Categories 2 and 3 represent pRab10 vesicles.
  k. Use *Calculate properties* block for each vesicle category. Calculated properties include number of vesicles, mean intensity, total intensity, and morphological properties.
    Representative images corresponding to the steps described above are shown in Figure 6 (panels a–i), with each panel corresponding to the respective step.

16. Use Harmony software building blocks to define the image analysis protocol.
  a. Use block *Filter image* in the Alexa 647 channel using Method: Smoothing, Filter: Gaussian.
    ***Note:*** The Gaussian smoothing filter is an image-processing step applied to improve image quality by reducing high-frequency image noise while maintaining the overall fluorescence signal pattern. The filtered image is returned as a new analysis channel (*Gaussian Smoothed*) for use in subsequent building blocks.
  b. Identify the cell-containing region in the Gaussian Smoothed channel.
    ***Note:*** The *Find image region* block defines regions of interest (ROI) within the image. A common pixel intensity threshold is applied automatically to detect bright areas relative to the surrounding background. A minimum area threshold is also applied to exclude objects smaller than cells (e.g., debris).
    ***Note:*** The common threshold is automatically set at 0.5 but can be adjusted from 0 to 1 depending on cell type and experimental conditions. Lower values increase the detected region; higher values reduce it.
  c. Use building blocks *Calculate morphology properties* and *Calculate intensity properties* in the identified region. Use the *Select population* block to isolate the population of interest (cells).
    ***Note:*** The measured properties include perimeter, area and Alexa 647 signal intensity. Regions that do not meet the defined selection criteria are discarded; included regions are shown with yellow borders.
  d. Use block *Find nuclei* (Method B) within selected image region, using channel Hoechst 33342. Set thresholds for minimum pixel intensity and minimum area to define valid nuclei. Subsequently, select *Calculate morphology properties* for detected nuclei.
    ***Note:*** Method B is chosen for robust detection of nuclei, particularly in images with high background signal.
    ***Note:*** The intensity threshold (0.40) and area threshold (>30 μm^2^) are optimized for the antibody and cell type used.
  e. To identify pRab10-positive vesicles in EGFP channel, use *Filter image* block with Method: Sliding Parabola (curvature 4).
    ***Note:*** The Sliding Parabola filter estimates and removes smooth, continuous background intensity while preserving punctate fluorescent signals. The image is separated into background and foreground components, and the foreground image is returned as a filtered image. This enhances the contrast of vesicular structures relative to the surrounding background and facilitates vesicle detection (see Figure 5 for a schematic of the filtering process).

**Figure 6:**
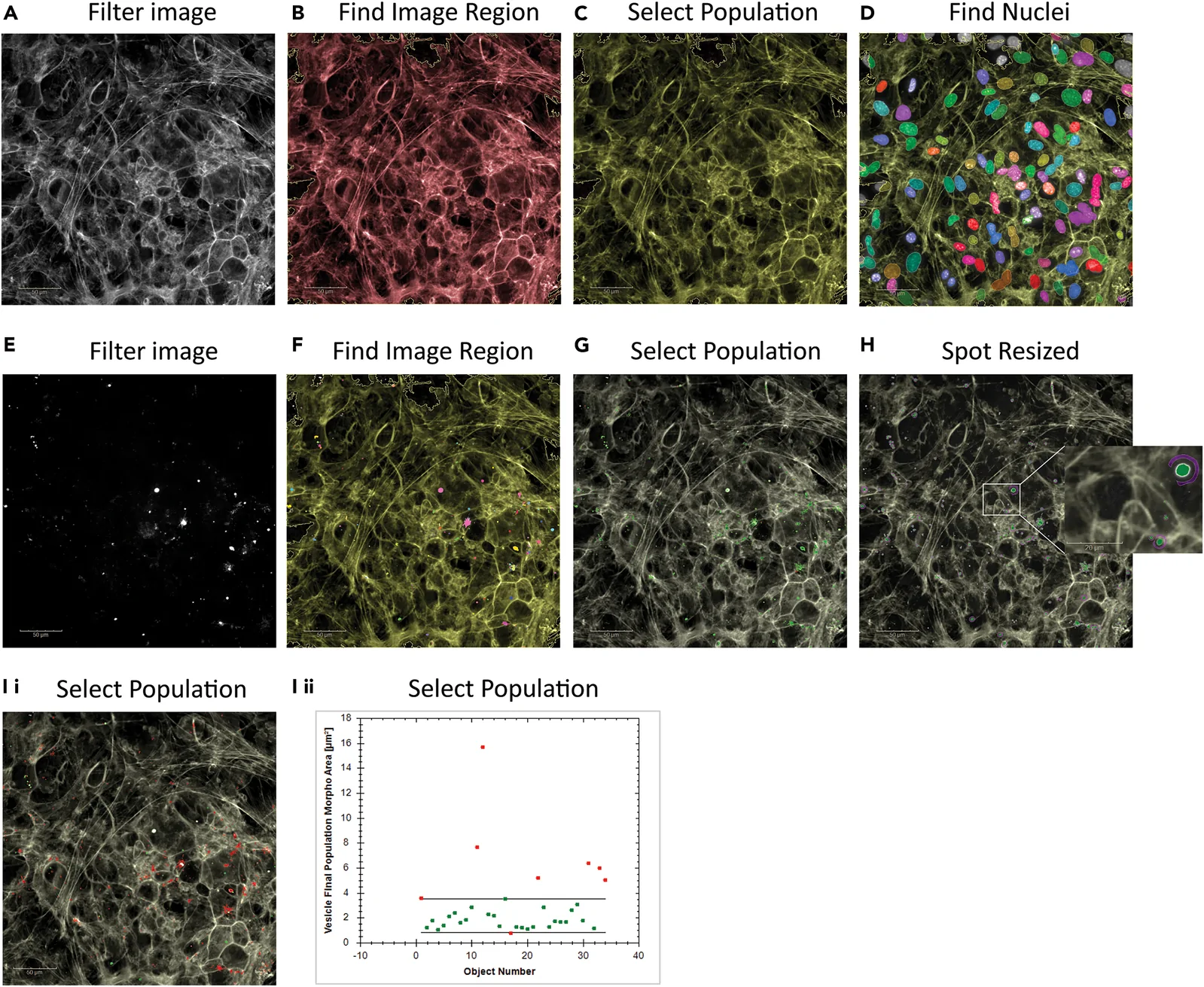
Representative images illustrating the building blocks described in Steps 16a–16i Images correspond to the sequential analysis workflow outlined in the protocol. Panel i.ii highlights one of the populations selected based on area criteria, as described in Step 16j. Scale bar 50 μm.
17. Run the protocol in Harmony software and export the results as an Excel file.

### Data analysis

This section describes the analysis and presentation of data generated using the Harmony software image analysis protocol.

18. Analyze data using GraphPad Prism (GraphPad Software, Boston, Massachusetts USA, www.graphpad.com).
  a. MLi-2 experiment: Use Equation: log(inhibitor) vs. response -- Variable slope to calculate IC_50_.
    ***Note:*** The Variable slope model fits the Hill slope from the data rather than assuming a standard slope. The x-axis represents the logarithm of inhibitor concentration, and the y-axis represents the number of pRab10-positive vesicles (Figure 7).
    **Figure 7:**
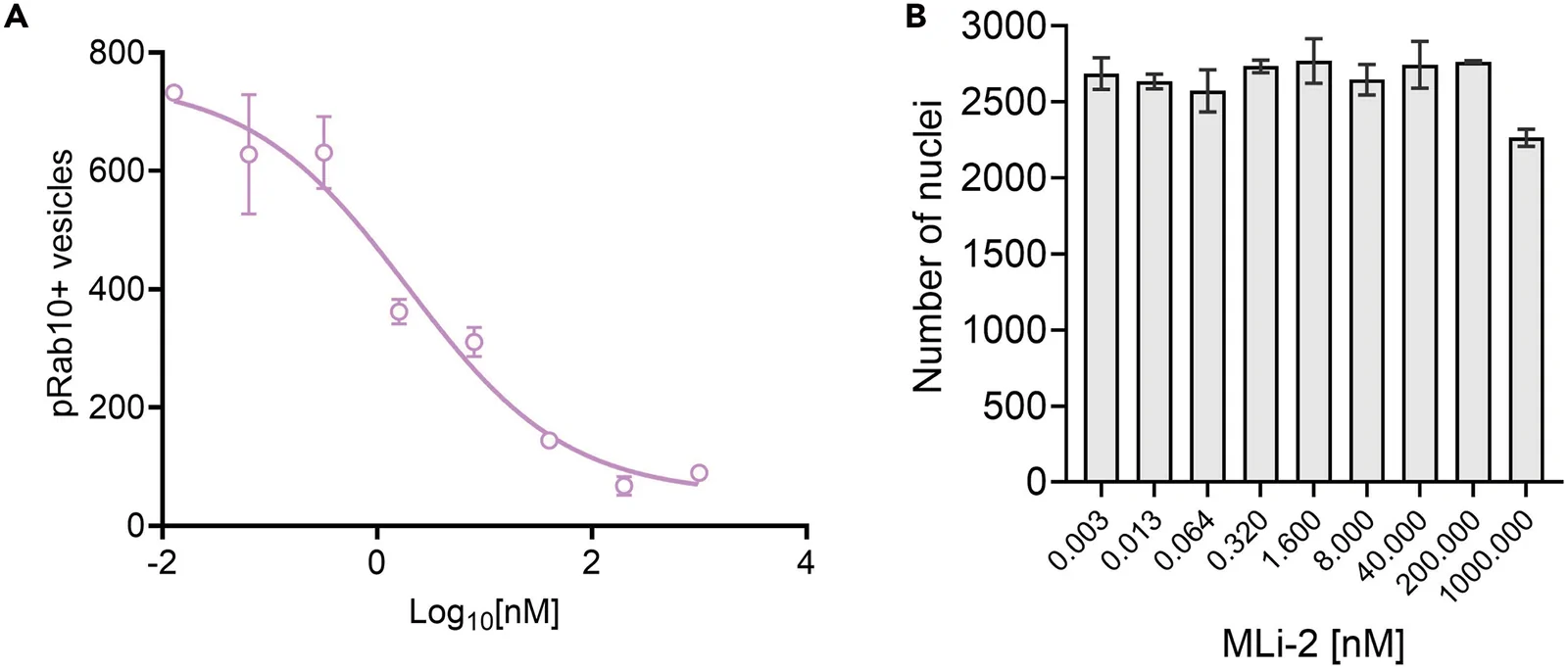
Dose–response analysis of LRRK2 inhibition by MLi-2 (A) Dose-response curve using LRRK2 inhibitor MLi-2 serial dilutions. Points represent mean ± SEM. IC_50_ = ∼3 nM. (B) Quantification of the number of nuclei per well across MLi-2 concentrations, demonstrating consistent cell numbers across treatment conditions. Data are shown as mean ± SEM.
  b. LLOME experiment: Data are presented as grouped bar graphs (Figure 9).

## Expected outcomes

MLi-2 is expected to produce a dose–response curve with an IC_50_ in the low nanomolar range (1–3 nM) (Figure 7). Previous studies have reported MLi-2 IC_50_ in a purified LRRK2 kinase assay in vitro (IC_50_ = 0.76 nM), a cellular assay monitoring dephosphorylation of LRRK2 pSer935 LRRK2 (IC_50_ = 1.4 nM), and a radioligand competition binding assay (IC_50_ = 3.4 nM).^11^ Although an IC_50_ for MLi-2 based on pRab10 levels has not been directly reported, the strong correlation between LRRK2 inhibition and reduction of Rab10 phosphorylation has been extensively documented in several cell types,^21^ including mouse primary astrocytes.^4^ Therefore, MLi-2 IC_50_ obtained from pRab10 measurements in this study can be confidently compared with the previously reported IC_50_ values for MLi-2.

**Figure 8:**
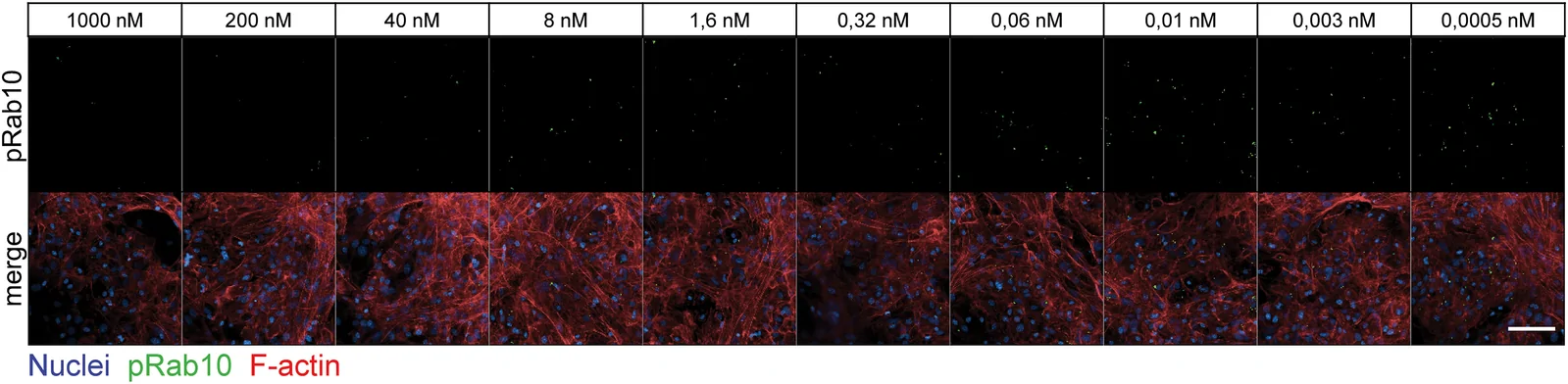
Concentration-dependent reduction of pRab10-positive vesicles following MLi-2 treatment Representative images acquired from a single field of view at the indicated MLi-2 concentrations, illustrating the concentration-dependent decrease in pRab10-positive vesicles. Scale bar 100 μm.

Visually, a progressive decrease in pRab10-positive vesicles is expected at higher MLi-2 concentrations. Figure 8 shows representative images acquired from a single field of view across the indicated MLi-2 concentrations.

**Figure 9:**
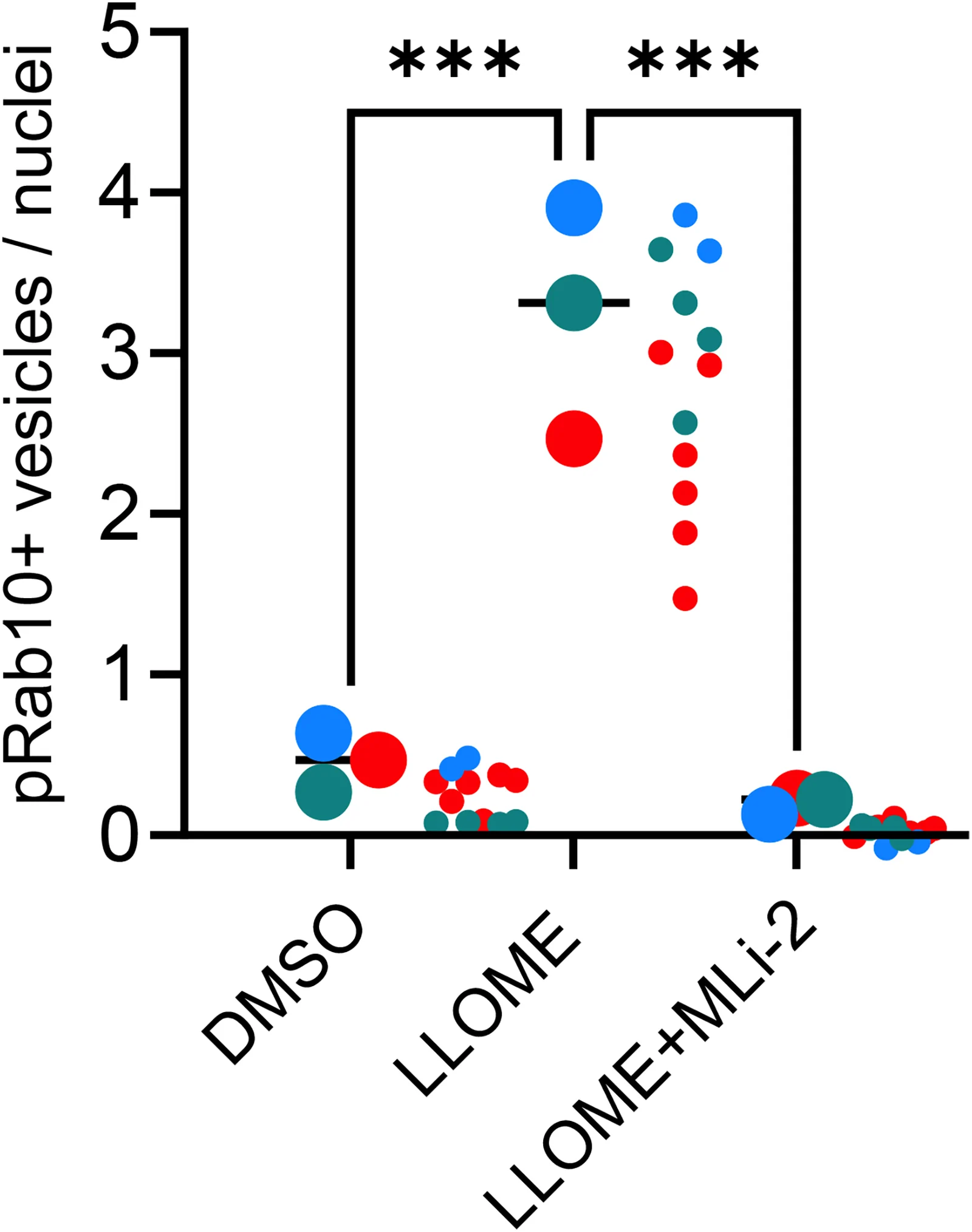
Effect of LLOME treatment on pRab10-positive vesicles and rescue by MLi-2 Quantification of pRab10-positive vesicles normalized to nuclei in *Lrrk2* G2019S primary astrocyte-enriched cultures treated with DMSO, LLOME (1 mM), or LLOME plus the LRRK2 kinase inhibitor MLi-2 (100 nM). Large circles indicate the mean value of each independent biological experiment; colors indicate the three independent biological experiments. Small dots shown next to each large circle represent individual technical replicates. Black horizontal lines indicate the mean of the biological replicates. Statistical analysis was performed on biological replicate means using one-way ANOVA followed by Tukey’s multiple comparisons test. ∗∗∗p < 0.001.

Treatment with the lysosomal stressor LLOME is expected to strongly increase the number of pRab10-positive vesicles.^12^ This increase should be rescued by co-treatment with MLi-2, as previously demonstrated in both human embryonic kidney 293 (HEK) cells^12^ and mouse embryonic fibroblasts (MEFs).^22^ These results are shown in Figure 9 and were quantified using high-content imaging.

## Limitations

This protocol is optimized for the Operetta CLS high-content imaging system described here. Although comparable automated imaging platforms may also be suitable, these were not tested in this study and adjustment of acquisition and analysis settings may be required for other systems. This protocol was developed using primary cortical astrocyte-enriched cultures carrying the pathogenic G2019S substitution within the endogenous murine *Lrrk2* gene. While it may be adapted to astrocytes derived from other brain regions or other cell types, differences in cell yield, morphology, and baseline LRRK2 activity may require optimization of seeding density, antibody selection (e.g., alternative structural marker) and imaging parameters (e.g., pRab10 signal intensity). Moreover, the culture preparation is not astrocyte-purified. The proportion of astrocytes and contaminating glial populations may vary between preparations, particularly depending on the efficiency and standardization of microglia-removal steps. For applications requiring highly purified astrocytes, additional standardized depletion/enrichment steps, such as immunopanning, cell sorting, or clodronate-microglia depletion should be incorporated and validated. Importantly, the modular structure of the protocol is intended to facilitate adaptation across different experimental contexts by providing clearly defined steps and baseline parameter settings that can serve as a starting point for systematic optimization. While automated nuclear counting provides an approximate measure of cell viability, primary astrocytes frequently exhibit multinucleation; therefore, nuclear counts do not directly reflect absolute cell numbers. Additionally, this assay does not include an evaluation of cell morphology. Confidence in cell number estimation could be improved by combining nuclear counts with cytoplasmic or cytoskeletal markers (e.g., phalloidin or cytoplasmic fluorescent dyes) to enable whole-cell segmentation. Assessment of changes in cell morphology could be incorporated by measuring total fluorescence intensity from these markers.

## Troubleshooting

### Problem 1

Low cell viability or altered cellular morphology (Step 1).

### Possible cause

Primary astrocyte-enriched cultures have undergone excessive passaging, which can result in altered morphology (e.g., elongated cells) or overgrowth of other glial subtypes.

### Potential solution

Use cells at early passages only. Seed cells at passage 1 and no later than passage 2 to maintain optimal morphology and viability.

### Problem 2

Uneven cell distribution across wells (Step 1).

### Possible cause

μClear 96-well plates have a slightly convex well surface (curvature of ∼200 μm), which can promote cell clustering toward the center of the well during seeding.

### Potential solution

Ensure the cell suspension is well mixed immediately before seeding. After seeding, leave the plate undisturbed at RT for approximately 10 min to allow cells to evenly distribute before transferring to the incubator.

### Problem 3

Altered cell morphology observed after immunostaining, characterized by lifted or folded cell edges.

### Possible cause

Excessively harsh washing steps may induce partial cell detachment, resulting in lifted and folded cell edges (Step 3–5).

### Potential solution

Perform washing steps gently by slowly adding and removing solutions along the side of the well. Avoid direct pipetting onto the cell layer and minimize aspiration strength to preserve cell adhesion.

### Problem 4

High background signal.

### Possible cause

• Insufficient washing between antibody incubation steps (Step 9–11).
• Aggregates in primary or secondary antibody solutions (Step 8, Step 10).

### Potential solution

• Increase the number or duration of washes between incubations.
• Centrifuge antibody solutions briefly before use to remove aggregates.
• Optimize antibody concentrations as needed to reduce non-specific staining.

## Resource availability

### Lead contact

Further information and requests for resources and reagents should be directed to and will be fulfilled by the lead contact, Elisa Greggio (elisa.greggio@unipd.it).

### Technical contact

Technical questions on executing this protocol should be directed to and will be answered by the technical contacts, Beatrice Masotti (beatrice.masotti@unipd.it) and Paolo Lorenzon (paolo.lorenzon.1@unipd.it).

### Materials availability

This study did not generate new unique materials.

### Data and code availability

This study did not analyze/generate new datasets/code.

## Acknowledgments

This work was supported by the 10.13039/100000864Michael J Fox Foundation for Parkinson Research (Grant nos. 023427 and 022431); by the European Union, NextGenerationEU (NRRP, Mission 4, Component 2, Investment 1.1, PRIN, Project 20222LRHCW, CUP C53D23005560006); and the Microscopy Core Facility at the Department of Biology, 10.13039/501100003500University of Padova.

## Author contributions

E.G. conceived the project. B.M., P.L., and G.C. wrote and optimized the protocol. B.M. and P.L. designed and performed the experiments and quantified the results. G.C. provided technical support. B.M. and E.G. wrote the manuscript. All authors helped with editing.

## Declaration of interests

The authors declare no competing interests.
