# Supplementary material for: Protocol to quantify LRRK2-mediated Rab10 phosphorylation using high-content imaging
Source: STAR Protoc. 2026 Sep 4;7(3):104819. doi: 10.1016/j.xpro.2026.104819 (PMC13571431; doi:10.1016/j.xpro.2026.104819)
Supplement: Supplementary file 1 [file mmc1.pdf]

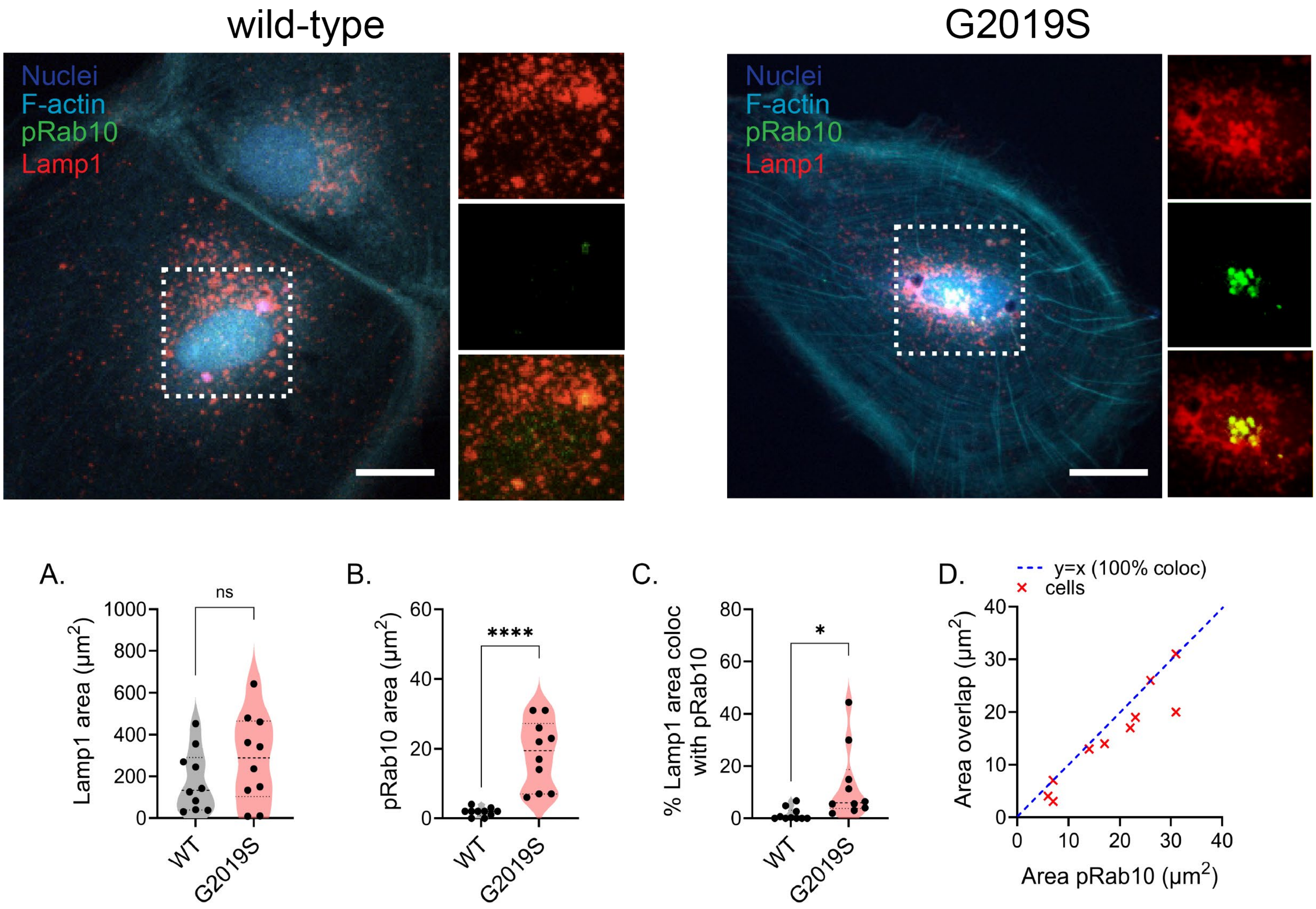

Supplementary Figure 1. Association of pRab10-positive structures with lysosomal compartments. Representative immunofluorescence images showing nuclei, F-actin, pRab10 staining and lysosomal marker LAMP1 in wild-type or *Lrrk2* G2019S primary astrocyte-enriched cultures. Quantification of fluorescence signal is shown in (A) LAMP1-positive area, (B) pRab10-positive area, and (C) percentage of LAMP1-positive area overlapping with pRab10 signal. (D) Percentage of pRab10-positive area overlapping with LAMP1-positive lysosomal structures in G2019S cells. Data are presented as individual cells. Statistical comparisons were performed using unpaired t-tests. Images were acquired using a Zeiss LSM 900 Confocal Microscope with a 40× objective. Scale bar: 20 μm
